# RE: A Clinical Decision Tool to Calculate Pretest Probability of Sentinel Lymph Node Metastasis in Primary Cutaneous Melanoma

**DOI:** 10.1245/s10434-023-13812-w

**Published:** 2023-07-03

**Authors:** Peter A. Prieto, Matthew S. Goldberg, Brian Martin

**Affiliations:** 1 Division of Surgical Oncology, Department of Surgery, Wilmot Cancer Center, University of Rochester Medical Center, Rochester, NY USA; 2 Department of Dermatology, Icahn School of Medicine at Mount Sinai, New York, NY USA; 3 Castle Biosciences, Inc., Friendswood, TX USA

Sentinel lymph node biopsy (SLNB) is a prognostic surgical procedure used to identify patients at highest risk of dying from melanoma, as part of the 8th edition of the American Joint Committee on Cancer (AJCC) staging criteria (AJCCv8). Patients with ≥5% risk of having a positive SLN are considered for (T1a with high-risk features/T1b) or offered (T2–T4) SLNB.^1^ However, using the T category from AJCCv8 (based exclusively on lesion Breslow thickness and ulceration status) to determine SLN eligibility has its limitations, as evidenced by the many algorithms and nomograms developed to incorporate additional clinicopathologic factors into the decision of whether to perform or avoid SLNB. Importantly, many of these nomograms have been shown to provide no benefit over SLNB for all patients, or have wide confidence intervals, reducing confidence in the prediction.^2–5^

Tripathi and colleagues describe another clinicopathologic-based tool for SLN metastasis prediction, i.e., ELMO (evaluating lymphatic metastasis outcomes).^6^ ELMO was developed and internally validated using data from the Surveillance, Epidemiology, and End Results (SEER) program and the National Cancer Database (NCDB).^6^ We concur with the authors that tools that can appropriately inform SLNB decision making are clinically useful.^7^ However, a limitation of the SEER database pre-2018 is that nodal status does not describe ‘SLN status’, but rather any ‘nodal status’, no matter how it was determined (e.g., palpation, fine-needle aspiration, or SLNB).

Underscoring this concern, the data used to develop ELMO do not appear consistent with published literature on stage-specific SLNB rates and suggest that 50% of patients with a known SLNB status enrolled in the SEER registry had a T1a tumor (Table 1), potentially resulting in a poorly calibrated model. To test the ability of ELMO to accurately predict SLN risk, we used ELMO (accessed using the online tool) to perform an analysis of T1b–T2a tumors with a known positive SLNB result, from a previously published dataset.^8^ Of the 41 SLN-positive patients analyzed, ELMO considered 24% (10/41) as having <5% risk, nearly five times higher than the accepted 5% false-negative rate.^1^

Finally, the authors state that “this personalized probability [of ELMO] is not available from any current commercial tests”. However, one such test that combines gene expression profiling (31-GEP) with clinical and pathological factors has demonstrated accuracy and precision for predicting SLN positivity, with 95% sensitivity and 98% negative predictive value,^8,9^ and the i31-GEP for SLNB was shown to provide net benefit in T1–T2 tumors over SLNB for all participants.^5,10^

There are clear limitations to the current staging system for determining the risk of SLN positivity, as reflected by the number of studies aiming to develop a better method for risk assessment. Why has the melanoma field failed to adopt a single clinicopathologic nomogram for SLNB metastasis risk? When will the field realize the shortcomings of algorithms that use only clinicopathologic factors? While we continue to evaluate the range of nomograms put forward for academic interest,^4,5^ tools incorporating GEP technology with clinicopathologic factors are supported by strong evidence and are clinically available to inform risk-aligned management for SLNB decision making.
